# Multiple Representations of Space by the Cockroach, *Periplaneta americana*

**DOI:** 10.3389/fpsyg.2018.01312

**Published:** 2018-07-30

**Authors:** Matthew B. Pomaville, David D. Lent

**Affiliations:** Department of Biology, California State University, Fresno, CA, United States

**Keywords:** vision, olfaction, allocentric memory, egocentric memory, visual snapshot, insect

## Abstract

When cockroaches are trained to a visual–olfactory cue pairing using the antennal projection response (APR), they can form different memories for the location of a visual cue. A series of experiments, each examining memory for the spatial location of a visual cue, were performed using restrained cockroaches. The first group of experiments involved training cockroaches to associate a visual cue (CS—green LED) with an odor cue (US) in the presence or absence of a second visual reference cue (white LED). These experiments revealed that cockroaches have at least two forms of spatial memory. First, it was found that during learning, the movements of the antennae in response to the odor influenced the cockroaches’ memory. If they use only one antenna, cockroaches form a memory that results in an APR being elicited to the CS irrespective of its location in space. When using both antennae, the cockroaches resulting memory leads to an APR to the CS that is spatially confined to within 15° of the trained position. This memory represents an egocentric spatial representation. Second, the cockroaches simultaneously formed a memory for the angular spatial relationships between two visual cues when trained in the presence of a second visual reference cue. This training provided the cockroaches an allocentric representation or visual snapshot of the environment. If both egocentric and the visual snapshot were available to the cockroach to localize the learned cue, the visual snapshot determined the behavioral response in this assay. Finally, the split-brain assay was used to characterize the cockroach’s ability to establish a memory for the angular relationship between two visual cues with half a brain. Split-brain cockroaches were trained to unilaterally associate a pair of visual cues (CS—green LED and reference—white LED) with an odor cue (US). Split-brain cockroaches learned the general arrangement of the visual cues (i.e., the green LED is right of the white LED), but not the precise angular relationship. These experiments provide new insight into spatial memory processes in the cockroach.

## Introduction

The cockroach’s environment is composed of a variety of sensory cues that convey important information about food, shelter, and danger. As the cockroach navigates through this sensory milieu it must be able to retain behaviorally relevant information. The utilization of internal and external cues facilitates the formation of proper associations about the relevant information, thereby maximizing the cockroach’s fitness. Integration of multimodal information and associative memory systems can function to signal spatially relevant information. This spatial information is assumed to be stored in the cockroach’s brain and used to facilitate the localization of objects and places. It has been shown that the cockroach uses both olfactory and visual spatial information to localize relevant goals in its environment (Lent, 2006). The cockroach uses of the spatial structure of an odor stimulus for directional orientation (Hösl, 1990) and the ability to learn the spatial relationship between visual cues (Mizunami et al., 1998; Kwon et al., 2004).

The results from experiments examining associative and spatial learning in restrained cockroaches reveal that the manipulations made to the sensory conditions under which cockroaches are trained can influence the nature of the resulting memory (Kwon et al., 2004; Lent and Kwon, 2004; Pintér et al., 2005; Lent et al., 2007). The results of these experiments characterizing associative learning (Lent and Kwon, 2004; Pintér et al., 2005; Lent et al., 2007) and spatial learning (Kwon et al., 2004) suggest that the cockroach may be using an unidentified spatial frame of reference to localize behaviorally relevant information. Using the antennal projection response (APR) assay to study associative learning and memory (Lent and Kwon, 2004; Pintér et al., 2005) revealed that the duration of the memory depends on the sensory conditions under which cockroaches are trained. Lent and Kwon (2004) showed that following training using non-restricted sensory conditions, the memory for the association persisted for at least 72 h, thus indicative of long-term memory (Lent and Kwon, 2004). However, cockroaches that were trained to associate sensory information presented to the antenna and the eye on one side only (restricted sensory condition) demonstrated APRs that persisted for less than 24 h (Pintér et al., 2005), suggesting a failure to consolidate the association to long-term memory. However, when taken into consideration with the results of other experiments (Kwon et al., 2004; Lent et al., 2007), it may suggest that the way in which cockroaches were trained resulted in two different memories being established; one for the general association of the cues and one for the spatial location of the cue. Kwon et al. (2004) and Lent et al. (2007) revealed that the sensory conditions that cockroaches experience during learning affected memory for the position of the CS and support the hypothesis that different memory are being established. Kwon et al. (2004) looked at responses to the CS at positions other than the trained position. In this experiment, APRs were elicited only when the CS was within 15° of the learned position which is close to the angular sensitivity of the cockroach in dark-adapted conditions (Heimonen et al., 2006) and it was suggested that the failure to show APRs toward these other positions may be due to the CS becoming ambiguous when moved in the environment. It has also been shown that following training with restricted sensory input, the APRs elicited from the side that did not receive odor or visual input during training were similar to the APRs elicited from the side that was trained and the memory was determined to be generalized (Lent et al., 2007). We hypothesize that these experiments are looking at two different types of memories. Cockroaches are either forming a memory for the simple association between two cues that is generalized or forming a memory that is for the spatial location of the cue and the way in which the antennae interact with the environment is important in determining which of one of these memories is formed.

In addition to better understanding how the sensory conditions result in the establishment of spatial memories, the APR should be further explored in conjunction with the split-brain cockroach assay (Lent et al., 2007). By combining the split-brain assay with a modified version of the spatial learning assay (Kwon et al., 2004), we would have an assay that could be used in the future to characterize the role of central structures, such as the central complex, and lateral structures, such as the mushroom bodies in spatial learning and memory (e.g., Mizunami et al., 1998). The mushroom bodies have long been shown to be involved in learning and memory (Heisenberg, 2003), and several studies have shown them to be important for visual and olfactory spatial behaviors. The mushroom bodies have been linked to spatial behaviors in cockroaches (Mizunami et al., 1998), butterflies (Montgomery et al., 2016; van Dijk et al., 2017), ants (Stieb et al., 2010, 2012; Grob et al., 2017), foraging in honey bees (Farris et al., 2001), and thigmotaxis in *Drosophila* (Besson and Martin, 2005), but their role in spatial learning and memory could be better characterized. Other spatial memory processes are either bilaterally distributed or involve the central complex (Ofstad et al., 2011; Seelig and Jayaraman, 2013, 2015; Martin et al., 2015; Varga and Ritzmann, 2016; Dewar et al., 2017; Turner-Evans et al., 2017).

Discussed here are a series of new experiments using modifications of established paradigms to reveal different types of spatial memory in the cockroach. We aim to test four hypotheses: (1) The memory for the spatial location of a visual cue is made more precise when the cockroach is able to freely sample its environment with both antennae during the learning of a visual–olfactory association. (2) The movement of the antennae in response to the odor source is providing the spatial information that helps to establish a spatial frame of reference during the learning of a visual–olfactory association. (3) The cockroach can simultaneously store spatial memory representing the angular relationship between two visual cues and spatial memory for a single visual cue learned relative to the odor spatial frame of reference. (4) The cockroach can establish a memory for the angular relationship between two visual cues using only half a brain. From these experiments, two types of representation of spatial information are considered: (1) spatial cues that are represented in relation to the cockroach, and (2) spatial cues that are represented in relation to each other (Benhamou and Poucet, 1998). Here, we suggest that the cockroach uses the spatial information, derived from olfactory and motor/proprioceptive feedback from paired antennae movements, to learn cues with respect to its own body. This olfactory sampling/antennal movement-derived egocentric memory and, previously identified spatial memory for positional visual cues, i.e., allocentric or visual snapshot memory, can both be used to localize a cue in space. Additionally, we demonstrate that the cockroach can form a memory for the relative position of two visual cues with half a brain. The results of these behavioral experiments provide a foundation to further explore the localization of spatial memory processes in the brain of the cockroach.

## Materials and Methods

### Animals

Experiments were conducted in Tucson, Arizona from 2005 to 2007 (Experiments 1 and 2) and in Fresno, California from 2012 to 2014 (Experiments 2 and 3) on adult male American cockroaches (*Periplaneta americana*) purchased from Carolina Biological Supply. The colony was maintained at approximately 25–28°C on a 12:12 light–dark cycle at 50–60% humidity. Rearing cages were supplied with natural cat food (Taste of the Wild Pet Foods, Meta, MO, United States or IAMS, Dayton, OH, United States) and natural peanut butter (JIF Natural, The J.M. Smucker Company, Orrville, OH, United States or Skippy; Bestfoods, Co., Englewood Cliffs, NJ, United States). Individuals with damaged or missing appendages or antennae were rejected for testing.

After 48 h of food deprivation and isolation, animals were anesthetized using CO_2_ and loaded into restraint tubes made from small polyethylene test tubes. The animals were secured with their heads and antennae exposed using a small dental wax collar, and the rear of the tube was sealed with laboratory parafilm. Animals secured in the restraint tubes were then placed into the testing room under red light and were left for at least 1 h to allow for recovery from the anesthetic. After the recovery period, the restrained animals were observed for natural antennae and leg movements. Animals displaying normal sampling (i.e., normal responses to air current, tactile stimulation, etc.) and a complete range of movement were moved into the training/testing arena for experimentation. In experiments using intact brain cockroaches, approximately 80% demonstrated normal antennal movement, and in the split-brain experiments, approximately 60% demonstrated normal antennal movements.

### Split-Brain Cockroach

Animals to undergo split-brain lesioning procedure were prepared as described by Lent et al. (2007). Cockroaches were anesthetized with CO_2_ and then restrained on a cold plate with their head immobilized using dental wax. An incision through the head capsule was made approximately 2 mm deep and 1–1.5 mm in length using a small razor blade in a blade holder. The incision was sealed using a droplet of melted dental wax and the animals were allowed 48 h in isolation to recover. Following behavior experiments, split-brain cockroaches were dissected and the extent of lesion characterized. Only those cockroaches that had their brain completely split, with the exception of the sub-esophageal ganglia, were included in the analysis.

Non-lesioned control animals were anesthetized using CO_2_ and were restrained on a cold plate in the same manner as animals undergoing the lesioning procedure. The control animals then had a drop of hot wax applied to the head in the same location as the lesion in non-control animals. Control animals were also placed in isolation cages for 48 h after the mock lesioning to prepare them for training and testing.

### Arena

#### Experiments 1 and 2

**FIGURE 1:**
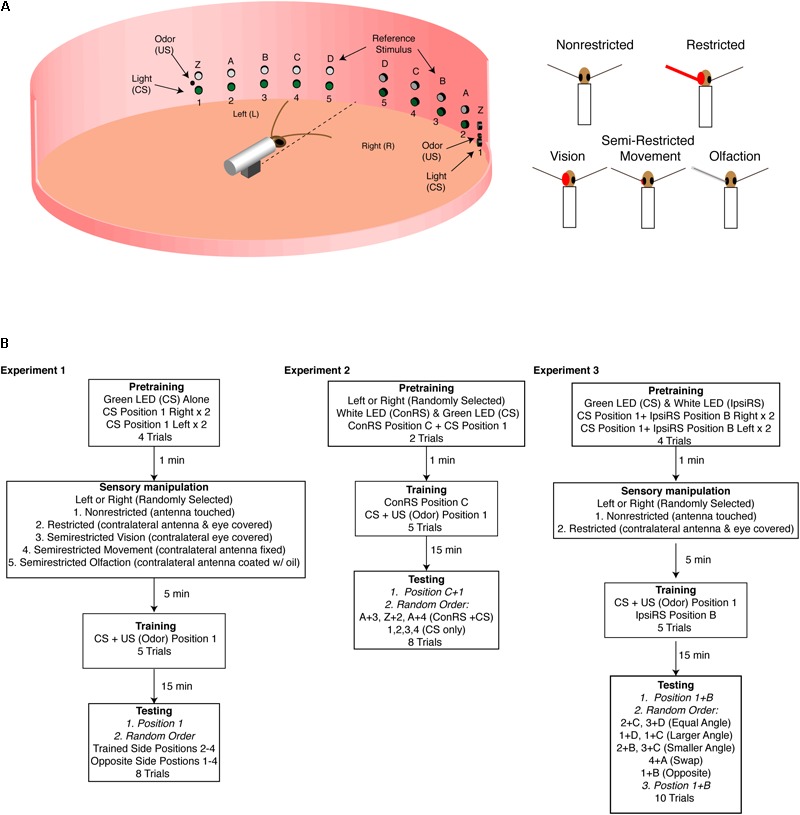
Experimental arena. **(A)** Diagram of the training arena with cockroach placed at the center (not to scale). A series of green LEDs and white LEDs are positioned starting 15° from midline extending to 75° at 15° intervals. At position 1 (left and right) are coincident cues odor source [unconditioned stimulus (US) and green LED conditioned stimulus (CS)] which are used during training. The white LEDs are used as the reference stimulus in Experiments 2 and 3. The sensory conditions are illustrated on the right and show the non-restricted, restricted (antenna and eye blocked), semirestricted vision (eye blocked), movement (antenna immobilized at the base), and olfaction (antenna covered with a thin film of oil). **(B)** Experimental protocols for the three experiments. In all the experiments, CS was 2 s and the US 1 s. In Experiments 2 and 3, which used a reference stimulus either on the contralateral side (Experiment 2) or the ipsilateral side (Experiment 3), the reference stimulus was always on unless otherwise noted. In Experiment 2, the reference stimulus (Z, A–D) is always presented on the side opposite of training—contralateral reference stimulus (ConRS). In Experiment 3, the reference stimulus is always presented on the same side as training—ipsilateral reference stimulus (IpsiRS).

As described in previous accounts (Kwon et al., 2004; Lent and Kwon, 2004), experiments were conducted in an arena enclosed within a visually uniform chamber illuminated with an infrared lamp. A restrained cockroach was positioned in the middle of the arena and aligned with respect to the green LEDs on the arena wall positioned at 15° intervals to the right and left of the insect (**Figure 1A**). The distance from the insect’s head to the position of these cues was 15 cm. Each LED was given a number, 1–5. Five white LEDs (E1000, Gilway Technical Lamp, Co., Woburn, MA, United States) were positioned on the wall of the arena to the right and left of the insect. These contralateral reference stimuli (ConRS) were also spaced at 15° intervals with respect to the cockroach and named Z, A–D. Food odors controlled by a solenoid valve were presented through an odor delivery system positioned at green LED 1. Stimuli and their sequences were controlled by a Grass S88 stimulator (Grass Instrument, Co., Quincy, MA, United States). In all experiments, the US was presented for 1-s and the CS for 2-s using simultaneous conditioning. A ventilation system was placed above the arena to remove odor after each trial (see Lent and Kwon, 2004 for details).

#### Experiment 3

The arena used was based on the design used by Kwon et al. (2004) and Lent and Kwon (2004) with some modifications to allow for multiple testing angles to be explored. The arena was formed using a 30 cm diameter wooden ring with vertical pairs of green (520–525 nm, 20,000 mcd, and C-LEDs) and white (6,000K, 20,000 mcd, and C-LEDs) LEDs every 15° from the centerline to 75° off center (**Figure 1A**). At the 75° position, a small polyethylene tube attaches to a syringe filled with an odor source (JIF Peanut Butter). Pure air puffs (charcoal filtered; air pressure 1 atm; and stimulus duration 1 s) were blown through the syringe cartridge containing the odor using a solenoid-controlled air source. All timing of lights and odor is done with a pair of Velleman MK188 Pulse-Pause timers (Velleman NV, Legen Heirweg 33, B-9890 GAVERE, Belgium, Europe). In all experiments, the US was presented for 1-s and the CS for 2-s using simultaneous conditioning. The odor concentration being delivered was only measured by observing behavioral responses. Permanent air flow was provided by an exhaust fan system placed above and behind the arena to remove odors from the inside of the arena between trials, and the surface of the arena cleaned with ethanol.

### Experiment 1: Training in Non-restricted, Restricted, and Semi-Restricted Sensory Conditions

Using the protocol and statistical analysis described by Lent and Kwon (2004) and Lent et al. (2007), intact brain cockroaches were conditioned with either non-restricted, restricted, or semi-restricted sensory input (**Figure 1A**). In all conditions, the protocol consisted of two pretraining trials of a two-second presentation of the green LED to both the left and right side of the animal at position 1 for a total of four pretraining trials (see **Figure 1B**) with a 1-min interval. Pretraining measured the cockroaches’ baseline response to the conditioned stimuli (CS). Animals which showed APR to the CS in all trials were rejected from further trials. Fewer than 10% of cockroaches elicited APRs to the CS in all pretraining trials.

After pretraining, the cockroaches were randomly trained to either position 1 on the right or position 1 on the left. Training comprised five trials of the green LED (CS) paired with the food odor, the unconditioned stimulus (US), as described by Lent and Kwon (2004). After the training was completed, animals responding to three or more presentations (60–72%) were isolated under a black cup for 15 min before testing to allow for the memory to be represented in a way that the APR could be elicited on the side opposite of the trained side (Lent et al., 2007). After 15 min, cockroaches were tested for the presentation of the CS at positions 1–4 on both the trained side and the opposite side in a random order. The CS was presented for 2 s, and APRs were measured for 30 s. The time interval between tests was 1 min.

If cockroaches were conditioned with non-restricted sensory input, both antennae could freely move and sample the olfactory environment. Additionally, they did not have any visual obstruction to the eye opposite of training (**Figure 1A**). If cockroaches were conditioned with restricted sensory input, the antenna on the opposite side of that given the CS + US pairing was secured with wax at the base and covered with a capped polyethylene tube. Additionally, the eye on the opposite side was covered with opaque wax (**Figure 1A**). Semi-restricted sensory conditioning involved three assays which either blocked visual input, antennal movement, or olfactory input on the opposite side of that given the CS + US pairing (**Figure 1A**). The semi-restricted sensory input assays were designed to examine the role of different sensory modalities. The first assay involved restricting only visual input to one eye while permitting antennal input. The second assay restricted proprioceptive reafferent sensory input by fixing the base of the antenna with wax and thus restricting movement of one antenna while allowing olfactory input. The third restricted olfactory input by covering the antenna with a thin film of light mineral oil (Fisher Scientific) allowing the animal to move its antenna, while reducing (<30% APR) significantly the ability of that antenna to sample odor (oil vs. normal response to odor: *n* = 15, *z* = 1.55, and *P* = 0.0128).

### Experiment 2: Training in the Presence of a Contralateral Reference Stimulus

Cockroaches were trained to associate an odor cue (US) with a green LED (CS) in the presence of a white LED reference stimulus on the contralateral side (ConRS) using the protocol described by Kwon et al. (2004) (**Figure 1B**). During training, the ConRS was on throughout the trial, unless otherwise noted. Training comprised two pretraining trials with the ConRS at position C, and the CS at position 1 to replicate the procedure described by Kwon et al. (2004). This was followed by five training trials of the ConRS and CS + US at positions C and 1, respectively. After the training was completed, animals responding to three or more presentations (65%) were isolated under a black cup for 15 min before testing. The testing phase comprised eight presentations of the CS and ConRS: two trials presented the ConRS and CS where the angular relationship was the same as training (C and 1; A and 3), two trials presented the ConRS and CS with angular relationships different from training (Z and 2; A and 4), and four trials of the CS alone in the absence of the ConRS at positions 1, 2, 3, and 4 (see **Figure 1**). The CS was presented for 2 s and APRs were measured for 30 s. Between each trial, the cockroach was covered with a black cup so that the ConRS position could be changed. The time interval between tests was 1 min. With the exception of the first test, which was always at the trained position, test position order was randomized.

### Experiment 3: Training in the Presence of an Ipsilateral Reference Stimulus (IpsiRS)

Intact brain and split-brain cockroaches were trained to associate an odor cue (US) with a green LED (CS) in the presence of a white LED reference stimulus displaced 30° medially on the ipsilateral side (IpsiRS) (**Figure 1B**). During training, the IpsiRS was on throughout the trial, unless otherwise noted. The protocol consisted of four pretraining trials with the IpsiRS at position B and the CS at position 1, presented two times to each side. Following pretraining, cockroaches were randomly assigned to the non-restricted or restricted group. The restricted group had the eye and antenna on one side, side randomly selected, covered. Cockroaches were given five training trials pairing the IspiRS and CS + US at positions B and 1, respectively. After the training was completed, animals responding to three or more presentations (60% intact brain and 46% split-brain) were isolated under a black cup for 15 min before testing.

Testing comprised 10 total trials. Nine trials were presentations to the trained half and one was to the naïve half. Tests to the trained half included: (1) three trials testing the APR when angular relationship between the IpsiRS and CS was the same as training, (2) two trials when the angular relationship between the IpsiRS and CS was greater than training, (3) two trials when the angular relationship IpsiRS and CS was smaller than training, and (4) one trial when the positions of the lights were swapped. Tests to the naïve half included: one trial testing the APR when the angular relationship is maintained, but mirrored, to test if the memory was generalized in a way that the cockroach remembered the IpsiRS was located anteriorly to CS. Due to the length of the testing period, all tests concluded with a trial testing the APR toward the original trained position on the trained half. This was to ensure that any lack of response was not due to fatigue or extinction of the learned response. Because of the time required to change the positions of the visual cues, 2-min intervals between tests were used. With the exception of the first and last test, which was always at the trained position, test position order was randomized. Finally, an experiment was done with intact brain cockroaches that were trained in the non-restricted sensory condition and tested at the trained position and angle but rotated to the contralateral side. In this rotated test to the contralateral side, the white LED (IpsiRS) was at position A and the green LED (CS) was at position 4 (**Figure 1A**). This was done to test if the response to the angular relationship was maintained even when rotated to the opposite side.

### Data Collection and Statistics

Data were collected through direct observation of the APR in restrained cockroaches as viewed through a video feed. To aid in accuracy, the floor of the arena had marks at each at ±3° CS position to give a window to score the APRs similar to that defined by Kwon et al. (2004). APRs were measured and analyzed as described by Kwon et al. (2004), Lent and Kwon (2004), and Lent et al. (2007). In each trial, cockroaches were given 30 s to respond to the stimulus. Only if the first movement of APR was directed toward the odor source location or to the CS (±3°) was it scored as a “1.” If the cockroach’s APR was toward a position other than the CS, if the cockroach struggled in the restraint during the stimulus, or if the antenna did not move from baseline in response to the stimulus, the response was scored as a “0.” The results from experiments were analyzed using non-parametric statistics. The Freidman’s test, Wilcoxon Signed-Rank test, or Wilcoxon Rank Sum test was used to identify significant difference from the pretrained response rate and differences between tests. The *F*-test of equality of variance was used to analyze the timing of contralateral antennal recruitment in intact and split-brain cockroaches. All statistical tests were run using MATLAB 2017A (Mathworks, Inc.).

## Results

### Visual–Olfactory Associations Reveal an Underlying Spatial Component

**FIGURE 2:**
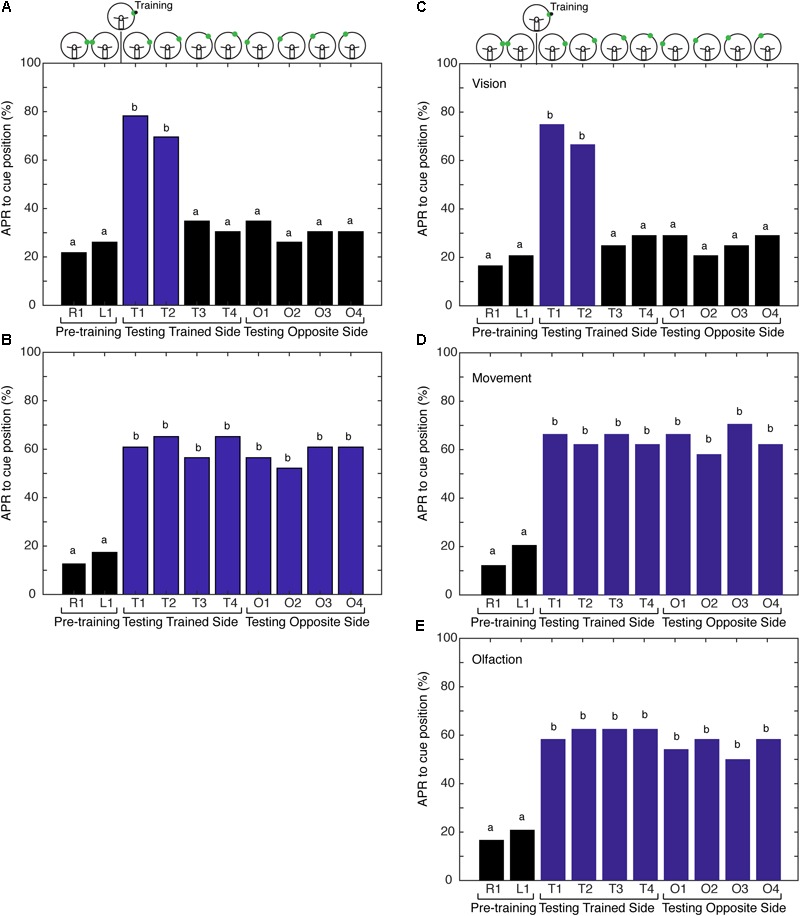
Comparison of antennal projection responses (APRs) using non-restricted, restricted, and semirestricted sensory conditions. **(A)** The APRs of cockroaches (*n* = 23) that were trained in the non-restricted sensory condition and tested on the trained side (T1–T4) and the opposite side (O1–O4). The pretraining APRs were not significantly different (*z* = –0.327, *P* = 0.7437). The APRs were significant when presented at the different trained positions (T1–T4) (χ^2^ = 27.15, *P* = 5.46E^-6^). Cockroaches demonstrated significant APRs when the CS was presented within 15° of the learned position (T1 and T2) (χ^2^ = 33, *P* = 3.23E^-7^), with no differences in the response to T1 vs. T2 (*z* = 0.6498, *P* = 0.5158). APRs were not different from pretraining when tested at other positions (T3 and T4 – χ^2^ = 6, *P* = 0.116; O1–O4 – χ^2^= 4, *P* = 0.2615). **(B)** APRs of cockroaches (*n* = 24) trained in the restricted sensory condition. The pretraining APRs were not significantly different (*z* = –0.3883, *P* = 0.6978). The APRs toward the CS were significantly different from pretraining (χ^2^ = 79.64, *P* = 1.9E^-13^), but were not different from each other at any of the positions (T1–T4 – χ^2^ = 12.35, *P* = 0.0895 and O1–O4 – χ^2^ = 8.68, *P* = 0.1223). **(C)** The APRs of cockroaches (*n* = 24) trained in the vision semi-restricted sensory condition. The pretraining APRs were not significantly different (*z* = –0.351, *P* = 0.7258). The APRs were significantly different between the tests (χ^2^ = 68.44, *P* = 3.045E^-12^). The APRs to the CS that was within 15° of the learned position (T1 and T2) were different from pretraining (χ^2^ = 135.95, *P* = 7.7E^-8^), but not each other (*z* = 0.6154, *P* = 0.5383). APRs were not significant at other locations (T3 and T4 – χ^2^ = 3.0, *P* = 0.2232; O1–O4 – χ^2^ = 7.3333, *P* = 0.1193). **(D)** APRs of cockroaches (*n* = 24) trained in the antenna movement semi-restricted sensory condition. The pretraining APRs were not significantly different (*z* = –0.7505, *P* = 0.4529). The APRs elicited to the CS positions were significantly different from pretraining (χ^2^ = 91.5, *P* = 8.14E^-16^), but were not different from each other (T1–T4 and O1–O4; χ^2^ = 11.2, *P* = 0.1301). **(E)** APRs of cockroaches (*n* = 24) trained in the olfaction semi-restricted sensory condition. The pretraining APRs were not significantly different from each other (*z* = –0.351, *P* = 0.7258). The APRs elicited to the CS positions were significantly different from pretraining (χ^2^ = 73.59, *P* = 3.00E^-12^), but were not different from each other (T1–T4 and O1–O4; χ^2^ = 13.18, *P* = 0.0679). Bar colors and letters reflect statistical groups. Illustration above graphs represents the position of cues during experiment.

The first hypothesis tested was that the memory for the spatial location of the visual cue is made more precise when the cockroach is able to freely sample its environment with both antennae during the learning of a visual–olfactory association. First, we examined if the association was generalized to the contralateral side of cockroaches that were conditioned with non-restricted sensory input and CS + US at position 1. The APRs were measured from the same side as training (trained half) at position 1 and the opposite side of training (naïve half) at position 1. APRs elicited from the “naïve half” of cockroaches were not statistically different from those elicited from untrained cockroaches (*n* = 18, Signed-Rank, *P* = 0.5) and, thus, the memory was not generalized. To examine the hypothesis of precision due to the presence of spatial information versus ambiguity due to movement of the CS in the environment, additional experiments were performed. Cockroaches were trained with either non-restricted or restricted sensory input to associate a visual cue and an olfactory cue. The training cues were offset 75° right from the midline. For testing, the cues were at positions 75, 60, 45, and 30° (positions 1, 2, 3, and 4, respectively), both right and left of midline. In the non-restricted sensory condition, APRs elicited following training were significantly different when the CS was presented at positions 1 and 2, but not at the other positions or on the contralateral side (**Figure 2A**). In the restricted sensory condition, APRs elicited following training were significantly different from pretraining. However, the APRs elicited from each individual position were not significantly different from each other (**Figure 2B**).

### Antennae Sampling Behavior Provides a Spatial Frame of Reference

Next, we tested the hypothesis that the movement of the antennae in response to the odor source delivered during conditioning is providing spatial information, establishing a spatial frame of reference during training. To address the underlying role of sensory processing in providing spatial information resulting in an APR that is spatially localized, the cockroaches were conditioned using paradigms that provide varying degrees of sensory restriction. This is designated here as conditioning with semi-restricted sensory input. Cockroaches trained under the first semi-restricted sensory condition (vision) elicited APRs to visual cues at different positions in a similar fashion to cockroaches trained under non-restricted sensory conditions. APRs were elicited only when the visual cue was tested at positions 1 and 2 and did not elicit APRs to a visual cue presented on the side opposite of that trained (**Figure 2C**). As visual input is already restricted by the design of the paradigm to one hemifield [outside of the binocular region (Seelinger and Tobin, 1981)], this result was not unexpected. It also demonstrated that the presence of the eye shield itself and any mechanical feedback that it may convey was not interfering with learning and memory processes in this assay. Cockroaches trained under both the second (movement) and third (olfactory) semi-restricted sensory conditions demonstrated APRs that were similar to cockroaches trained under restricted sensory conditions. When only the movement of the antenna was blocked, the cockroaches elicited APRs toward visual cues irrespective of where the cue was positioned (**Figure 2D**). APRs toward visual cues were significantly different from pretraining at all positions and APRs elicited at different positions were not significantly different from each other. Similarly, when only olfactory information was blocked cockroaches elicited strong APRs toward visual cues positioned in either hemifield (**Figure 2E**). These APRs were significantly different from pretraining, but not significantly different from each other.

**FIGURE 3:**
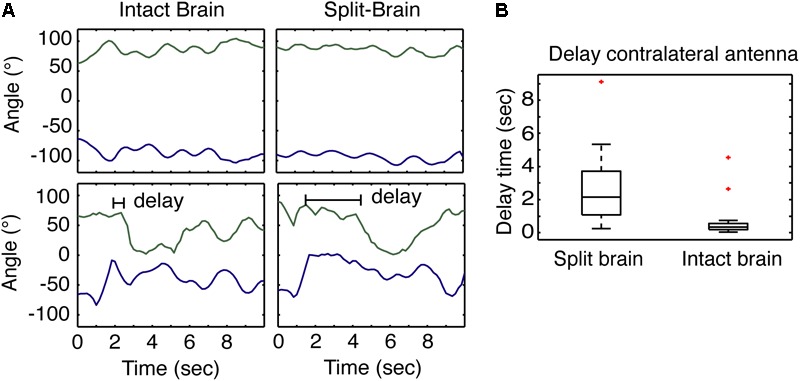
Antennae response to odors in split- and intact brain cockroaches. **(A)** Example of the at rest movement of the antennae of a restrained cockroach (top) compared to the response of both antennae of a restrained cockroach following odor stimulation displaced 45° to one side. **(B)** The response of the contralateral antennae in split-brain cockroaches (*n* = 24) was significantly delayed compared to the response of intact brain cockroaches (*n* = 30) (*F* = 5.18, *P* = 5.08E^-5^; *z* = 5.09; *P* = 3.48E^-7^).

The results of varying the degrees of sensory restriction during learning suggest recruitment of the contralateral antenna is important. To better understand how the contralateral antenna may be contributing to sampling the odor cue, we use the split-brain assay which has been shown to decouple antennal movements (Lent et al., 2007). Given that cockroaches only show spatially restricted APR to a single cue when both antennae are able to freely move and sample the odor, we hypothesized that non-restricted sensory conditioning results in the quicker recruitment of the contralateral antenna and the coupling of antennal movements that may provide the idiothetic cues necessary for the establishment of an egocentric spatial frame of reference. Here, we looked at the time to the recruitment of the contralateral antenna from the onset of odor and compared the response in split-brain (*n* = 24) and intact brain (*n* = 30) cockroaches. When recording the horizontal position of the tips of the antennae of restrained cockroaches at rest, the movements of intact brains are typically synchronous whereas those of the split-brain are asynchronous (**Figure 3A**, top). To test the recruitment of the contralateral antenna to an odor stimulus, cockroaches were presented with a single 2-s pulse of odor at the 45° position, and the time it took each antenna to begin sampling was measured (**Figure 3A**, bottom). The recruitment of the contralateral antenna was significantly delayed in the split-brain cockroaches compared to the intact brain cockroaches (**Figure 3B**).

### Parallel Memory Processes and Spatial Representations

**FIGURE 4:**
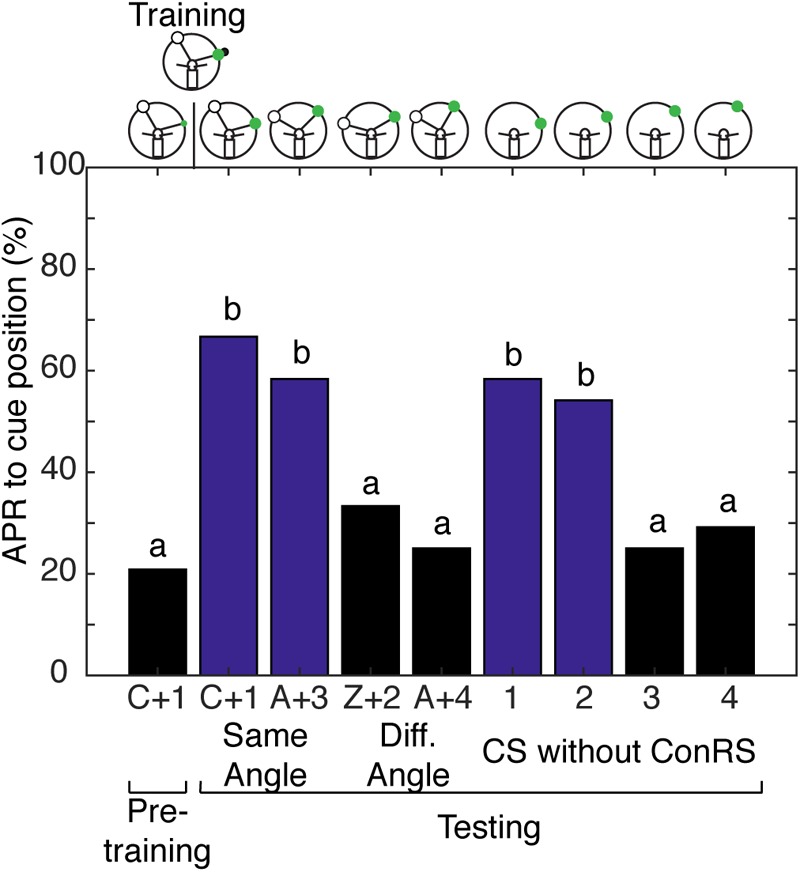
Antennal projection responses demonstrate multiple representations of space. The APRs of cockroaches (*n* = 24) that were trained in the presence of a contralateral reference cue were examined with different configurations of the ConRS and CS. When tested, the APR to the CS in the presence of a ConRS were significantly different (χ^2^ = 22.67, *P* = 4.739E^-5^). When tested at positions that maintained the ConRS–CS training relationship, APRs were significantly different from pretraining (χ^2^ = 18.73, *P* = 8.58E^-5^), but not different from each other (*z* = 0.5777, *P* = 0.5643). When tested with other configurations that had larger or smaller angular relationships the APRs were not significantly different from each other (*z* = 0.6154, *P* = 0.5383) or pretraining (χ^2^ = 4.67, *P* = 0.097). When tested without the ConRS and only the CS at positions 1–4, the APRs were significantly different from each other (χ^2^ = 20, *P* = 0.0002). There were significant APRs when the CS was presented within 15° of the learned position (1 and 2), but not when the CS was presented at other locations (3 and 4) (1 and 2 vs. pretraining, *n* = 24, χ^2^ = 16.23, *P* = 0.0003; 3 and 4 vs. pretraining, *n* = 24, χ^2^ = 3, *P* = 0.2231). The APRs to positions 1 and 2 were not significantly different from each other (*z* = 0.2759, *P* = 0.7826), nor were the APRs to positions 3 and 4 (*z* = –0.308, *P* = 0.7581). Bar colors and letters reflect statistical groups. The illustrations above the graphs represent the position of cues during the experiment.

With evidence to suggest cockroaches can localize a single cue in space when allowed to freely sample the environment, we tested the hypothesis that cockroaches can simultaneously store a spatial memory representing the angular relationship between two visual cues and a spatial memory for a single visual cue learned relative to the egocentric reference. Cockroaches learn to associate the CS + US in the presence of a contralateral reference visual cue (Kwon et al., 2004) but, do they also learn the CS using the egocentric frame of reference? To address this question, cockroaches were trained to associate a visual cue with an olfactory cue in the presence of a ConRS. The APRs of cockroaches were then tested both in the presence and absence of the reference cue. Cockroaches learned the spatial relationship between the ConRS and the CS similarly to those previously described (**Figure 4**). When the CS was presented at varying positions in the absence of the reference, however, they elicited APRs only in a limited region of space (**Figure 4**). Thus, cockroaches elicited APRs only if the CS was presented within 15° of the trained position. However, cockroaches would respond to the CS outside of this 15° range when the CS was coupled with the ConRS.

These findings lead to another question: can cockroaches learn just the angular relationship between two visual cues and not the egocentric-derived spatial representation? To address this question, cockroaches were conditioned with semi-restricted sensory input that blocked movement of, and olfactory input to the antenna on one side by covering it with a small tube, while permitting visual input to both eyes. These cockroaches were trained to associate the visual cue with the olfactory cue in the presence of a ConRS. Cockroaches trained under these conditions did not demonstrate either form of spatial learning; their APRs were similar to those classically conditioned with restricted sensory input. The APRs of cockroaches trained in this condition were significantly higher than pretraining (*n* = 24, χ^2^ = 16.4444, and *P* = 0.00248) and not significantly different from each other (*n* = 24, χ^2^ = 4.50, and *P* = 0.2123). Thus, cockroaches elicited APRs irrespective of where the CS was presented during the different tests.

### Spatial Learning Localized to Half of the Brain

**FIGURE 5:**
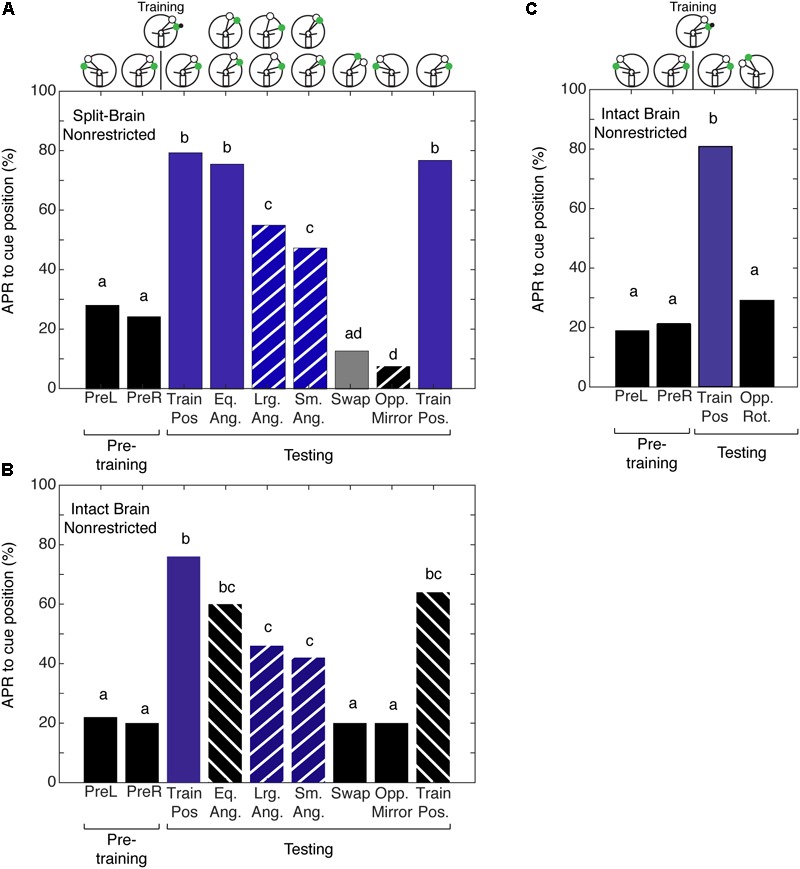
Antennal projection responses of split-brain and intact brain cockroaches trained in the non-restricted sensory condition unilateral spatial learning assay. **(A)** The APRs of split-brain cockroaches (*n* = 39) during pretraining were not significantly different (*z* = 0.5416, *P* = 0.5881). The APRs elicited during tests were significant (χ^2^ = 158.8, *P* = 1.321E^-29^). The first and last test APRs were different from pretraining (first test: *z* = –6.14, *P* = 8.23E^-10^, last test: *z* = –5.86; *P* = 4.58E^-9^), but not each other (*z* = 0.2655, *P* = 0.7906). APRs in tests that maintained the angular relationship were similar to each other (*z* = 1.304, *P* = 0.1923), different from pretraining (*z* = –7.18; *P* = 7.05E^-10^) and similar to the first and last test (*P* > 0.30). When tested at position with larger angular relationships, APRs were similar to each other (*z* = 0.2204, *P* = 0.8254), as was the APRs in tests at the smaller positions (*z* = –0.2195, *P* = 0.8262). The APRs in larger and smaller tests were not different from each other (*z* = –0.956, *P* = 0.03391), but were lower than the trained angle response (same vs. large: *z* = 2.68, *P* = 0.007; same vs. small *z* = –3.61, *P* = 3.1E^-4^) and greater than pretraining (large: *z* = –4.32, *P* = 1.52E^-5^; small: *z* = –3.22, *P* = 0.0012). APRs in the swapped and mirrored position tests were similar to or decreased from pretraining (Swap: *z* = 1.76, *P* = 0.078; Opp. Mirror: *z* = 2.48, *P* = 0.013). **(B)** The APRs of intact brain cockroaches (*n* = 25) during pretraining were not significantly different (*z* = 0.239, *P* = 0.811). The APRs elicited during testing were significant (χ^2^ = 69.8, *P* = 1.669E^-11^). The first and last test APRs were different from pretraining (first test: *z* = –5.25, *P* = 1.54E^-7^, last test: *z* = –4.19; *P* = 2.77E^-5^), but not different from each other (*z* = 0.9043, *P* = 0.3658). APRs in tests that maintained the angular relationship were similar to each other (*z* = 0.0, *P* = 1) and the first and last tests (*P* > 0.23), but greater than pretraining (*z* = –4.02, *P* = 5.73E^-5^). The APRs to larger angles were similar to each other (*z* = 0.2697; *P* = 0.7874) as were the APRs to the smaller angles (*z* = 0.2723, *P* = 0.7854). The APRs to the larger and smaller angular relationship were similar to each other (*z* = –0.397, *P* = 0.691) and greater than pretraining (Lrg. Ang.: *z* = –3.16, *P* = 0.0016; Sm. Ang.: *z* = –2.69, *P* = 0.0072), similar to the same angle tests (Eq. Ang. vs. Lrg. Ang.: *z* = 1.27, *P* = 0.204; Eq. Ang vs. Sm. Ang.: *z* = 1.6, *P* = 0.11) and decreased compared to the first trained position test (Lrg. Ang.: *z* = 2.443, *P* = 0.0145; Sm. Ang.: *z* = 2.757, *P* = 0.0058), but not the last test (Lrg. Ang.: *z* = 1.454, *P* = 0.458; Sm. Ang.: *z* = 1.778, *P* = 0.0754). APRs in the swapped and mirrored position tests were similar to pretraining (Swap: *z* = 0.9161, *P* = 0.105; Opp. Mirror: *z* = 0.9161, *P* = 0.105). **(C)** The APRs of intact brain cockroaches trained with non-restricted sensory input were not significantly different from each other during pretraining (*z* = –0.3527, *P* = 0.7243) and during testing were only different from pretraining to the first test using the trained positions of the CS + IpsiRS (1 + B: *z* = –3.831, *P* = 1.27E^-4^). When tested with the same angular relationship but rotated to the contralateral side, APRs were not significantly different from the pretraining (*z* = –0.47, *P* = 0.638) and significantly different from the other test position (*z* = 2.47, *P* = 0.0134). Bar colors and letters reflect statistical groups. Illustration above graphs represents the position of cues during experiment.

Given that we could not separate the two forms of spatial memory in the intact brain cockroach, we wanted to examine the limits of the cockroaches’ abilities to establish a memory for the angular relationship between two visual cues. To test this limit, we characterized spatial memory in the split-brain cockroach. We hypothesized that cockroaches can establish a memory for the angular relationship between two visual cues using only half a brain. Split-brain cockroaches (*N* = 39) that were trained with non-restricted sensory conditioning showed significant APRs toward the CS paired with the IpsiRS when the angular relationship closely matched that of learning (**Figure 5A**). When the angular mismatch between the CS and IpsiRS was too large or too small, the APR was significantly lower than the trained angle response but was also significantly different from the pretraining response. If the position of the CS and the IpsiRS were swapped, APRs were significantly reduced and were similar to those observed in pretraining. The cockroaches were given a single presentation of the mirrored cue combination to the side opposite training to check for possible memory generalization (i.e., white light is anterior to green light) as demonstrated by Lent et al. (2007) and cockroaches demonstrated a significantly lower APR toward the cue compared to pretraining. Finally, cockroaches were given a test at the original training position and they demonstrated a strong APR, suggesting that the memory was still intact and the prolonged testing procedure did not result in diminishing the response due to lack of reinforcement (**Figure 5A**). As a comparison, cockroaches that had an intact brain, but underwent mock surgery were trained. When intact brain cockroaches (*N* = 25) were trained using non-restricted sensory conditions, the responses to the trained angular relationship of intact brain cockroaches were similar to those observed in the split-brain cockroach. The cockroaches showed significant APRs when presented with the CS in the presence of the IpsiRS (**Figure 5B**). Again, similar to the split-brain cockroaches, the intact brain cockroaches elicited an APR to the CS paired with the IpsiRS at both larger and smaller angles. The percentage of APRs toward the larger and smaller CS and IpsiRS angular relationships was the less than as learned relationship, but greater than pretraining. If the position of the CS and IpsiRS were swapped, the APRs were reduced to pretraining levels, as they were when the CS and IpsiRS were presented mirrored to the side opposite of training (**Figure 5B**). Finally, we trained a group of intact brain cockroaches (*N* = 11) using non-restricted sensory condition to associate the CS + US in the presence of the IpsiRS and performed two tests. One test was with the CS and IpsiRS at the same position as training and one test with the same angular relationship as training but rotated to the contralateral side. Cockroaches showed significant APRs only at the trained position and not when the paired visual cues were rotated to the contralateral side (**Figure 5C**).

**FIGURE 6:**
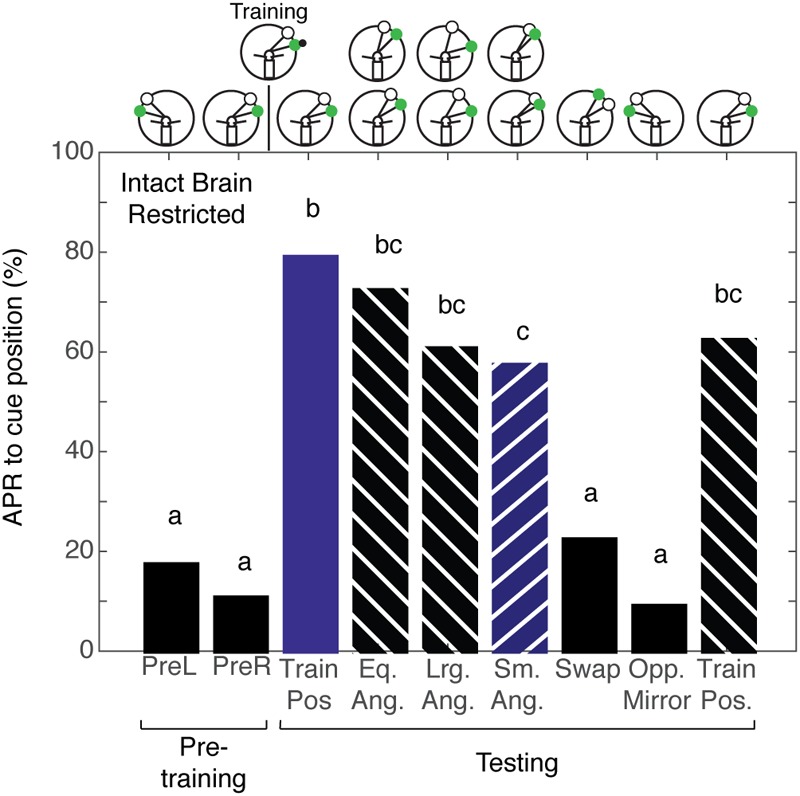
Intact brain cockroaches trained in the restricted sensory condition unilateral spatial learning assay. The APRs of intact brain cockroaches (*n* = 30) during pretraining were not significantly different (*z* = 1.014, *P* = 0.310). The APRs elicited during testing were significant (χ^2^ = 112.68, *P* = 4.18E^-20^). The first and last test APRs were significantly different from pretraining (first test: *z* = –7.07, *P* = 1.60E^-12^, last test: *z* = –5.47; *P* = 4.46E^-8^), but not each other (*z* = 1.411, *P* = 0.1582). APRs in tests that maintained the angular relationship were significantly different from pretraining (*z* = –7.74, *P* = 9.90E^-15^), but not each other (*z* = 0.5693, *P* = 0.5691) or from the trained positions tests (*P* > 0.26). The APRs to larger angles were similar to each other (*z* = –0.2545; *P* = 0.7991) as were the APRs to the smaller angles (*z* = –0.251, *P* = 0.8018). The APRs to larger angles were similar to each other (*z* = 0.368, *P* = 0.713), were greater than pretraining (Lrg. Ang. – *z* = –6.39, *P* = 1.69E^-10^; Sm. Ang. – *z* = –5.99, *P* = 2.04E^-9^), and similar to the same angle tests (Eq. Ang. vs. Lrg. Ang.: *z* = 1.355, *P* = 0.175; Eq. Ang vs. Sm. Ang.: *z* = 1.721, *P* = 0.085). The larger angle tests were similar to both the first (*z* = 1.739, *P* = 0.082) and last (*z* = 0.1478, *P* = 0.802) trained position test. APRs in the smaller angle tests were decreased compared to the first trained position test (*z* = 2.022, *P* = 0.0431), but not the last (*z* = 0.4488, *P* = 0.6535). APRs in the swapped and mirrored position tests were similar to pretraining (Swap: *z* = –1.089, *P* = 0.277; Opp. Mirror: *z* = –0.699, *P* = 0.4841). Bar colors and letters reflect statistical groups. Illustration above graphs represents the position of cues during experiment.

Given that cockroaches that are trained to associate the CS + US in the presence of a ConRS fail to establish a memory for the angular relationship when antenna is restricted, we wanted to test if the same was true for intact brain cockroaches trained to the CS + US in the presence of an IpsiRS. Intact brain cockroaches that were trained using restricted sensory conditions (*N* = 30) demonstrated APRs similar to the APRs of intact brain cockroaches trained using non-restricted sensory conditions. The APRs were significantly different from pretraining when the angular relationship was the same, larger, and smaller (**Figure 6**). Similarly, the APR toward larger and smaller CS and IpsiRS angular relationship was significantly different from pretraining. Only when the position of the CS and IpsiRS was swapped or mirrored on the opposite side did APRs reduce back to pretraining levels. Contrary to what is observed when using restricted sensory conditioning with no reference cues, the memory of the CS-IpsiRS was not generalized to the other side and there was not a significant APR when the CS and IpsiRS were presented on the opposite side of that which was trained (**Figure 6**).

## Discussion

### Odor Spatial Frame of Reference

These results, combined with previous accounts (Kwon et al., 2004; Lent and Kwon, 2004; Lent et al., 2007), suggest that cockroaches trained with a restricted sensory input elicit APRs to the visual cue regardless of its position, whereas cockroaches trained with non-restricted sensory input elicit APRs only in the trained hemifield and only if the visual cue does not deviate drastically from the learned position. From the new experiments described in this paper and those described earlier (Kwon et al., 2004; Pintér et al., 2005; Lent et al., 2007), two hypotheses regarding the memory of the learned visual–olfactory association can be developed. First, a single antenna processing olfactory stimuli is sufficient to associate an olfactory cue with a spatially coincident visual cue. In this assay, this association is generalized and the visual cue is indicative of an odor irrespective of where it appears in the environment. Second, both antennae sampling information from an odor source results in providing not only directional information but also positional information. The olfactory cue’s positional information is detected with respect to the cockroach itself, presumably because it bilaterally processes and integrates olfactory and motor/proprioceptive information. Varga and Ritzmann (2016) demonstrated that the cockroach, *Blaberus discoidalis*, encodes head direction using idiothetic cues in the absence of external cues. In our current work, the bilateral movement of the antenna during olfactory sampling may be providing the necessary idiothetic cues to establish the egocentric frame of reference. We hypothesize that the movement of the antennae results in the creation of an idiothetic frame of reference that can be used to learn the position of the CS relative to the cockroach. The possibility that there were any visual cues other than the green LED in the environment conveying spatial information, thereby creating additional visual landmark references, can be ruled out as all experiments were performed under infrared light conditions (a non-visible wavelength for cockroaches), and the training arena and surrounding area were visually uniform. Even though cockroaches are restrained and the retinotopic array of the eyes should be sufficient to provide all the spatial information needed, cockroaches only respond to the CS within a limited range if both antennae are able to move freely. We suggest that additional spatial information is being provided by the olfactory cue and the movement of both antennae in response to the odor which may help to reinforce the spatial information provided by the retinotopic organization of the input to the eyes. However, this needs to be further examined.

When analyzing the movements of antennae, the recruitment of the contralateral antenna provides additional insight into the behavioral response in the restrained assay. The baseline movements of the antenna are synchronous in the intact brain and asynchronous in split-brain restrained cockroaches in the absence of any delivered chemosensory or mechanosensory stimuli. Both synchronous and asynchronous movements in cockroaches are common. Cockroaches typically show stronger spatio-temporal coupling during walking rather than pausing (Okada and Toh, 2004). The increased spatio-temporal coupling observed in our assay (restrained = pausing) may be resultant of the design of the experiments, where cockroaches are restrained and not walking. When an odor stimulus was delivered, there were differences in the responses of the intact brain and split-brain cockroaches, with the contralateral side being recruited faster in intact brain cockroaches. The observations of antennal movements in the restrained condition suggest that coupling of antenna movements require bilateral and/or centralized control processes. In the split-brain cockroach, these control processes may be disrupted, and thus may affect recruitment of the contralateral antenna in response to an odor presentation. This early recruitment may be important in providing a spatial frame of reference and deserves further consideration.

### Multiple Spatial Memories

Cockroaches elicit an APR that are spatially constrained when they are conditioned with both antennae free to sample the olfactory environment. The cockroaches, simultaneously, represented space in terms of the angular relationships between visual cues. This visual snapshot memory for the angular relationship between the two cues provides a memory that allows the cockroach to elicit an APR when presented with a similar angular arrangement of the visual cues during tests. By using snapshot or image matching the cockroach can compare its current view with the memory for the angular relationship of the two cues and only elicit an APR if the overall image similarity is high (Zeil et al., 2003). Thus, the snapshot memory representing the angular relationship of two visual cues further contributes to the cockroaches’ ability to localize learned cues and has been proposed as a mechanism to facilitate visual navigation in insects (Collett and Collett, 2002). When both representations of space can be utilized by the cockroach to localize a learned cue, the memory for the angular relationship between the CS and reference stimulus must be the one that determines the behavioral response. This response may be unique to this particular behavioral assay. When olfactory sampling was blocked using semi-restricted sensory conditioning on the contralateral side of intact brain cockroaches, and they were trained to associate the angular relationship between the two cues (CS + ConRS), the cockroaches failed to learn the angular relationship. The cockroaches’ APRs following training in this condition were toward CS + ConRS angular relationships that were the same, smaller, and larger, as well as, to the CS alone at all positions. Interestingly, the cockroaches’ response to the CS + ConRS is similar to what we see in cockroaches conditioned to the IpsiRS + CS, which also elicit APRs to angular relationships that are the same, smaller, and larger than the trained angular relationship. The failure of the cockroach to learn the precise angular relationship of the CS + ConRS in this semi-restricted condition could be due to one of two things. First, the learning is sequential and formation of the memory for the angular relationship of the CS + ConRS requires the other spatial frame of reference to be established first, which cannot be done when the antenna is restricted. Second, the design of the experiment intricately links olfaction and vision in the training paradigm, such that the formation of both types of spatial memory requires bilateral olfactory processing. We believe that the restriction of the antenna is the constraining factor and bilateral processing results in increased precision when learning the location of the visual cues. While the two types of spatial memory can be experimentally separated, as demonstrated in the experiments described above, it is unlikely that the learning of the two spatial representations can be experimentally separated using this paradigm.

### Spatial Learning and Memory in Half a Brain

The APR of the split-brain cockroaches demonstrated the acquisition of a learned unilateral spatial association. The results of this testing show that in split-brain models, the angular association between the ipsilateral reference light and the conditioned light can elicit an APR, even if the position of the lights is changed relative to the position of the cockroach. Previous studies show that the split-brain cockroaches perform as well as intact brain cockroaches during conditioning of the APR (Lent et al., 2007). It is known that there should not be any rotation of the CS more than 15° from its original position because the APR will be diminished (Kwon et al., 2004). However, this performance is improved by coupling the CS with a reference cue (Kwon et al., 2004). A similar improvement is seen in the split-brain cockroach and most clearly demonstrated when the position of the light cues was swapped. Another key finding is that when intact brain cockroaches are unilaterally trained using a spatial learning protocol, if the paired light cues are rotated into the contralateral field the cockroaches no longer elicit APRs. Additionally, the generalization of the memory, as tested by a mirroring of the cues, from the trained side to the untrained side is blocked and while the response in this test is even less than pretraining, it is significantly lower compared to what is expected from a positive response, reflecting the expected non-response during presentation of the cues. Perhaps, the additional cues provided help by giving an additional frame of reference to the cockroaches, thus allowing them to learn on which side the odor should be expected when they encounter the light cues in their environment. The ability of the brain to learn unilaterally may be a general phenomenon, because it has been shown that, in honey bees, the two brain halves can learn quite different association tasks independently, if each side is shielded from the stimuli presented to the other side (Sandoz and Menzel, 2001). It was expected that the immobilized animal could achieve monocular spatial memory. However, we did not know if this would require the integrity of both brain halves or whether this could be achieved after midline sectioning. Earlier studies (Mizunami et al., 1998) showed that place memory is abolished only when both mushroom bodies are lesioned and can be achieved as long as one mushroom body on the same side is undamaged. Ofstad et al. (2011) demonstrated that the central complex is necessary for spatial learning and place memory and an increasing number of studies have shown that orientation, visual-guided behaviors, and landmark recognition depend on the central complex (Seelig and Jayaraman, 2015; Dewar et al., 2017; Stone et al., 2017; Turner-Evans et al., 2017). When all of our experiments are taken as a whole, the results provide support for the role of both central complex and mushroom bodies in spatial memory.

### Spatial Memory in the Cockroach

**FIGURE 7:**
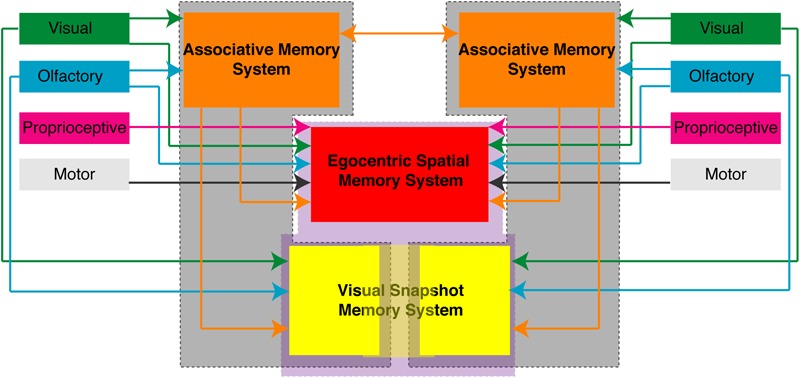
Proposed organization of memory processes in the brain of the cockroach. Based on the behavioral responses observed in restrained cockroaches trained in various associative and spatial learning assays suggest that different memory processes are unilaterally distributed (gray enclosed) or centrally/bilaterally distributed (purple enclosed). Associative memory process (orange) and some allocentric spatial memory processes (yellow) can function unilaterally. Egocentric spatial memory (red) processes and some visual snapshot or allocentric memory processes (light yellow) require central or bilateral processing. Shown are the sensory inputs and connections each of these areas makes that have been experimentally shown using the different APR behavior assays.

The APR assay can be used with varying degrees of sensory restriction in the intact brain cockroach or the split-brain cockroach and provides us with a number of behavioral protocols to examine associative and spatial learning and memory processes. Given the APR assay was designed to be used in a restrained cockroach, it provides a platform for electrophysiological studies which will allow us to better understand the neural basis of these behaviors. The organization of spatial memory and the dynamics of memory transfer still needs additional investigation in order to better understand how such processes are organized in the brain of insects. Importantly, the results of these and previously published (Kwon et al., 2004; Lent and Kwon, 2004; Pintér et al., 2005; Lent et al., 2007) experiments examining the APR in the restrained cockroaches suggest that these processes are distributed both unilaterally and bilaterally/centrally (**Figure 7**) which may provide us with brain areas to target.

Some experiments have been done to localize learning and memory processes to specific brain areas in *P. americana*. Associative memory processes are likely localized in the mushroom bodies and can be generalized from one side to the other over time (Lent et al., 2007). Mizunami et al. (1998) provided evidence for a neural basis of spatial learning in the cockroach. Cockroaches are able to use visual cues to learn the location of a hidden cool spot on a heated floor, but they have significantly reduced spatial learning and memory when the mushroom bodies have been lesioned bilaterally (Mizunami et al., 1998). This work suggests that spatial learning takes place either through communication between or convergence on the same output center of the paired mushroom bodies found in each brain hemisphere. A number of other studies have also provided evidence that the mushroom bodies may be important for spatial and visual behaviors. It has been shown that there is correlation between the plasticity of the mushroom body calyces and the size and spatial complexity of host range in the butterfly, *Polygonia c-album* (van Dijk et al., 2017) and spatial orientation is related to calyx expansion in *Helioconius* butterflies (Montgomery et al., 2016). The mushroom bodies may also be important for visual navigation in desert ants, *Cataglyphis fortis* (Stieb et al., 2010, 2012) and *Cataglyphis noda* (Grob et al., 2017), as well as foraging in honey bees (Farris et al., 2001; Lutz et al., 2012; Cabirol et al., 2018). In the fruit fly, *Drosophila melanogaster*, the mushroom bodies have been shown to distinctly segregate visual and olfactory sensory input (Vogt et al., 2016). A model looking at navigation of the desert ant, *C. fortis*, has shown that the mushroom body circuitry has the capacity to facilitate visual homing using snapshot matching (Ardin et al., 2016).

Many recent studies have focused on characterizing the role of central complex in spatial learning and memory and in navigation. In insects, the prominent midline structure comprising the ellipsoid body, fan-shaped body, protocerebral bridge, and noduli (Ito et al., 2014) has long been shown to be important for locomotor activity (Strauss, 2002; Strausfeld and Hirth, 2013). The central complex also plays an important role in the integrative behaviors such as visual orientation and spatial integration (Homberg et al., 2011). It plays a role in visual pattern memory during foraging behaviors of *D. melanogaster* (Liu et al., 2006; Wang et al., 2008), visual pattern recognition (Pan et al., 2009), and spatial learning and place memory (Neuser et al., 2008; Ofstad et al., 2011; Varga et al., 2017). Disrupting central complex processing in *D. melanogaster* impacts spatial learning and memory (Ofstad et al., 2011). The ellipsoid body, containing the ring neurons, are known to be important for the recognition of visual patterns (Seelig and Jayaraman, 2013), have been shown to respond to visual landmarks when available (Seelig and Jayaraman, 2015), and may provide the neural substrate for visual navigation (Dewar et al., 2017). Research is increasingly demonstrating the importance of the central complex in spatial behaviors and navigation, such as path integration and steering in the bee (Stone et al., 2017), internal representation of the heading in *D. melanogaster* (Kim et al., 2017; Turner-Evans et al., 2017). In another species of cockroach, *B. discoidalis*, the central complex has been shown to be important in coding head direction relative to both internal cues and landmarks (Varga and Ritzmann, 2016), in addition to context-dependent movement (Martin et al., 2015). When taking into account all of this research, there is evidence that suggest that the mushroom bodies, the central complex, and/or the integrity of the projections running through the central brain are essential to spatial learning and memory in many invertebrate species.

## Conclusion

The findings from the experiments presented here invite an interesting comparison between the spatial mapping in the cockroach and the parallel map theory of hippocampal function (Jacobs and Schenk, 2003; Jacobs, 2012). The parallel map theory proposes that the hippocampus encodes space with two mapping systems. One, the “bearing map,” encodes space based on directional cues such as gradients. The other, the “sketch map,” encodes space based on positional cues. While the findings from the cockroach demonstrate the possible existence of comparable spatial frames of reference, it remains an open question whether the cockroach is using the olfactory cue, specifically the gradient information provided by the odor plume as a spatial frame of reference and if this frame of reference is encoded in parallel with the visual snapshot. While behavioral comparisons of spatial mapping in the cockroach and in animals with a hippocampus, such as rats, are quite possible with the current learning experiments, attributing such mapping functions to any structure of the insect brain, as they have been for the hippocampus is an interesting challenge. These findings demonstrating spatial learning and memory capabilities of the cockroach, and the large amount of research increasingly showing that there are many similarities in the neural underpinnings of navigation and spatial behaviors in insects and mammals (Seelig and Jayaraman, 2015; Varga and Ritzmann, 2016; Kim et al., 2017; Turner-Evans et al., 2017; Varga et al., 2017), deserve further investigation and should invite further comparisons between spatial learning in mammals and insects.

## Data Availability

The raw data supporting the conclusions of this manuscript will be made available by the authors, without undue reservation, to any qualified researcher.

## Author Contributions

DL conceived, designed and performed the experiments, analyzed the data, and wrote the manuscript. MP performed the experiments, analyzed the data, and provided text and figures for the manuscript.

## Conflict of Interest Statement

The authors declare that the research was conducted in the absence of any commercial or financial relationships that could be construed as a potential conflict of interest.
